# Use of real-world evidence to support regulatory decisions on medical devices in China and a unique opportunity to gain accelerated approval in “Boao Lecheng Pilot Zone”

**DOI:** 10.1186/s12962-022-00412-w

**Published:** 2023-01-18

**Authors:** Jiahe Li, Lichang Liu, Haijun Cao, Mei Yang, Xin Sun

**Affiliations:** 1 Happy Life Tech, Boston, MA 02494 USA; 2 Happy Life Tech, Shanghai, 200233 China; 3 Happy Life Tech, Short Hills, NJ 07078 USA; 4 Chinese Evidence-based Medicine Center, and NMPA Key Laboratory for Real World Data Research and Evaluation in Hainan, West China Hospital, Sichuan University, Chengdu, 610041 China

**Keywords:** Real-world evidence, Real-world data, Medical device, China, Regulatory decision

## Abstract

This article aims to summarize the development and challenges of real-world data (RWD) and real-world evidence (RWE) in China and introduce a unique opportunity for medical devices to gain accelerated regulatory approval in China by utilizing RWE generated in a free trade pilot zone “Boao Lecheng” in Hainan Province. In 2020, the National Medical Products Administration (NMPA) issued a draft guideline on the “Use of real-world data to support clinical evaluation for medical devices”, suggesting that RWE derived from RWD could support clinical evaluation throughout the life cycle of a medical device. Meanwhile, the Chinese government has allowed qualified RWD collected in Boao Lecheng to support registration application of innovative medical devices and drugs in China. These medical devices and drugs should have been approved abroad, but not in China yet, and met urgent and unmet medical needs in China. The article also presents the successful story of an innovative Glaucoma drainage tube as the first medical device approved in China using RWE generated in Boao Lecheng in 2020. Although we are witnessing an increased interest in RWE, a few challenges remain, e.g., limited data accessibility and data sharing, concerns on data quality, etc. Collaborations among relevant stakeholders in the RWE research are vital to address the challenges.

## Introduction of medical devices and their registration process in China

The medical device industry in China is rapidly developing and relevant policies are increasingly evolving in recent years. Medical devices are defined as instruments, equipment, appliances, in vitro diagnostic products (and calibrators), materials, and other similar items used directly or indirectly on the human body, including pertinent computer software [1]. They play vital roles in disease prevention, diagnostics, and treatments.

The National Medical Products Administration (NMPA) (formerly the China Food and Drug Administration, or CFDA) categorizes medical devices into three classes according to their risks: Class I devices are low-risk devices that require general control, such as bandages and exam gloves; Class II devices are intermediate-risk devices that require strict control, such as suture needle and electrocardiograph; Class III devices are high-risk devices that require special strict control, such as implantable pacemaker and intravascular catheters [2, 3].

In China, the NMPA reviews medical device registration applications, and decides whether to approve the applications based on safety, effectiveness, and quality assessments. For imported Class I devices, applicants should provide relevant materials to the NMPA for records. For imported Class II or III devices, the NMPA reviews the applications, and issues “Medical Device Registration Certificate” after approval. The applicants of imported devices should provide proof of market approval in the countries (or regions) where the imported devices are registered or manufactured; however, for innovative medical devices, overseas market approval is not required. Additionally, three types of medical devices are eligible for special registration programs and could be prioritized in the registration process: innovative devices, devices for urgent and unmet clinical needs, and devices for public health emergencies [2, 4].

## Guideline on the use of real-world data to support clinical evaluation for medical device in China

Clinical evaluation of medical devices is essential in regulatory decision-making. With the development of real-world data (RWD) in research, RWD are progressively used in clinical evaluation throughout the life cycle of a medical device [5].

To standardize and guide the application of RWD in the clinical evaluation of medical devices in China, the NMPA issued a draft guideline on the “Use of real-world data to support clinical evaluation for medical devices” in 2020. The guideline defines RWD as “data pertaining to patient health status and/or the routine delivery of healthcare collected from a variety of sources, except for traditional clinical trials”. It also defines real-world evidence (RWE) as “the clinical evidence regarding the usage, and potential benefits or risks, of a medical product derived from analysis of RWD, which could be effective in supporting regulatory decision-making” [6].

The guideline emphasizes the quality of RWD, where data relevance and data reliability are two key elements of RWD quality assessment: the former means if the data are sufficient to answer all the clinical questions relevant to a given research objective; the latter relies on the accuracy of data collection. The NMPA assesses data quality from six specific aspects: representativeness, completeness, accuracy, authenticity, consistency, and repetitiveness. The sources of RWD include electronic medical records (EMRs) from hospitals, medical claims, medical device registries, and so on [6].

The guideline also suggests that RWE derived from RWD could support clinical evaluation throughout the life cycle of medical device, including premarketing and postmarketing clinical assessments. Some of the common scenarios where RWE could be utilized include supporting registration of innovative imported medical devices for urgent clinical use, modification of indications and contraindications, postmarketing surveillance, etc. These scenarios suggest that although RWE is increasingly utilized as supplementary evidence in medical device clinical evaluation, it cannot replace the present clinical evaluation pathway [6]. This echoes messages from similar guideline issued by the Food and Drug Administration or the European Medicines Agency [7, 8].

## Challenges of real-world evidence generation and potential solutions

With increased interest in the generation of RWE in China, a few challenges remain. The key one is limited data accessibility and data sharing. China’s nationally funded basic health insurance covers over 95% of the Chinese population; however, there is no public access to the medical claims data in the national social insurance system [9]. EMR systems are disconnected across different healthcare institutions in China, resulting in the fragmented patient information. Additionally, the ethical approval process for real-world studies using RWD is lengthy and difficult, and ethics review boards often question the potential benefits of these studies [10]. To address these concerns, the policy makers should establish explicit policies to increase data access and data sharing, strengthen privacy protection, and improve the ethical approval process [10]. A national integration and data sharing platform could be established and involve multi-stakeholder participation, including central and local governments, hospitals, health information technology enterprises and so on [11].

Another challenge is the accuracy, completeness, and consistency of data. There is a lack of systematic technical guidance for data collection or data quality control in China [10]. The quality and completeness of data vary greatly by hospitals and regions, and RWE research highly relies on elite hospitals in major cities in China. Longitudinal follow-up data are often inadequate, due to data disconnection across healthcare institutions [12]. For medical devices, product uses may be recorded in EMRs, while device-specific outcomes, such as calibration records or unique device identifiers, are usually not documented there. We should therefore encourage collaborations across all the relevant stakeholders to develop systematic technical guidance and improve data quality by types of data (e.g., EMRs, medical claims, registries).

The wide gap between the limited research capacity and the great demand also exists. Leading researchers with in-depth understanding of methodologies, data and healthcare settings in China are lacking [10]. Academic institutions should develop rigorous academic programs and foster the next generation of talents and leaders in the field of RWE research.

## Hainan Boao Lecheng Pilot Zone: a unique opportunity for innovative medical devices to gain accelerated approval in China

Although a few challenges exist in generating RWE, there is a growing interest in utilizing RWE for regulatory approvals. Recently, China has introduced a unique opportunity for medical devices to gain faster market access to China by leveraging RWE generated in a free trade pilot zone in Boao Lecheng, Hainan Province.

Since 2018, Chinese government authorities have issued a series of policies to guide the use of RWE in accelerating the market approval of innovative drugs and medical devices, which should have been approved abroad, and meet urgent and unmet (no replaceable products approved in China) medical needs in China [13–16]. In June 2019, “Hainan Boao Lecheng International Medical Tourism Pilot Zone” (pilot zone) was initiated by the NMPA and the Hainan Provincial Government [17]. In September 2019, the “Implementation Plan” of the pilot zone was issued by the National Development and Reform Commission, etc. The plan specified that qualified RWD collected in Boao Lecheng could support registration application of innovative drugs and medical devices [18]. This allows the most needed medical products to reach Chinese patients faster.

### Real-world data collection within and outside of Boao Lecheng

Boao Lecheng is currently the only region in China authorized to use innovative medical devices, which should have been approved abroad but not in China yet [19]. Patients usually travel to the pilot zone and utilize the devices as prescribed by physicians or through surgeries. Upon completing treatments in the pilot zone, patients could take the devices back home, and either regularly return for follow-ups, or have follow-ups in local hospitals [19].

Comprehensive information is collected through different data sources within and outside of Boao Lecheng (Table 1) [19]. The main data source is EMRs (such as data from diagnoses, lab results, and prescriptions) routinely collected from medical institutions in Boao Lecheng, while patients use innovative medical devices there. Other data sources could be data proactively collected outside of and within the pilot zone, including data from patients’ local medical institutions (e.g., past medical history, past diagnoses and treatments, and follow-up data after returning home), and data from Boao Lecheng medical institutions (e.g., adverse events, patient-reported outcomes, follow-ups). Proactive data collection could be retrospective (e.g., gathering of past medical history) or prospective (e.g., collecting outcomes in follow-ups), through interviews, phone calls, etc. [20]. Additionally, overseas registration information, wearable devices and the “Boao Lecheng Real-World Data Research Platform” are also valuable sources for RWD.

**Table 1:** Boao Lecheng innovative medical devices’ real-world data

Category	Data Sources	Collection Method	Information Collected
Medical data outside of Boao Lecheng	Patients’ local medical institutions	Proactive collection	Past medical history, past diagnoses and treatments, compliances, other follow-up data, etc.
Medical data within Boao Lecheng	Medical institutions in Boao Lecheng	Routine collection	Symptoms, diagnoses, imaging, lab results, prescriptions, other medical information, etc.
		Proactive collection	Adverse events, patient-reported outcomes, etc.
Data from past research of medical devices	Medical device manufacturers	Proactive collection	Overseas registration information, published data, etc.
Data collected from other devices or innovative platforms	Wearable devices	Routine collection	Heart rate, blood pressure, sleeping status, daily activities, etc.
	Boao Lecheng Real-World Data Research Platform	Routine collection	Product tracing and monitoring across entire life cycle of a medical device
	“Lecheng Health” smartphone app	Routine collection	Patient recruitment, patient registration, appointments, on-line consultation, product compliance, self-reported adverse events, etc.

Connecting, structuralizing, and standardizing data from the above sources provide a solid foundation of the “Boao Lecheng Real-World Data Research Platform”, which has gone live since May 2022 [21]. Relevant stakeholders could leverage the platform to support the life cycle evaluation of a medical device product—from registration to postmarketing adverse event surveillance.

### Common real-world study types in Boao Lecheng

Real-world studies in Boao Lecheng are different from those conducted in other regions for a few reasons: access to the medical devices is limited to the pilot zone; patients are from different regions with diverse background; uneven quality of evidence from overseas registration and postmarketing surveillance exists. Appropriate study design is critical to generate high quality evidence. A few types are often considered as below, which are also the common real-world study types outlined in the NMPA guideline [6, 22]:

- Observational study: depending on the quantity and quality of existing RWD (such as EMRs), observational studies could be prospective (with little or low-quality RWD), retrospective (with large amount of high-quality RWD) or ambidirectional (with some high-quality RWD).
- Single-arm trial with external control: the intervention group could include patients from medical institutes within or outside of Boao Lecheng. The external control group could be historical control or parallel control. Historical control includes data generated before the first patient is enrolled in the single-arm trial, e.g., previous RWD. Parallel control can be a patient registry built simultaneously with the single-arm trial.
- Single-arm trial or observational study comparing to objective performance criteria (OPC): for some rare or life-threatening diseases without any treatment in China, a single-arm trial or observational study is feasible. Outcomes from patients receiving a novel device in a one-arm trial could be compared to OPC, which refers to a numerical target value derived from guidance, expert opinions, historical data and so on [23].
- Pragmatic clinical trial: there are two types of pragmatic clinical trials depending on the location of the control group. Tthe first type enrolls patients in local medical institutes, after that the intervention group receives medical devices in Boao Lecheng while the control group receives standard of care locally. The second one recruits all the patients in Boao Lecheng, and both the intervention and the control groups receive corresponding treatments in the pilot zone.

### Glaucoma drainage tube—the first medical device approved using domestic RWE in China

XEN® (an innovative Glaucoma drainage tube) has been approved in over 30 countries to treat glaucoma, the most common irreversible blinding disease in the world impacting 22 million patients in China [24, 25]. In April 2019, XEN® entered China and received the permission to be used in Boao Lecheng to fulfill the unmet medical need in China [26]. One month later, the first glaucoma surgery using XEN® was conducted successfully in Boao Super Hospital. On March 26, 2020, XEN® was approved in China as the first medical device using domestic RWE for registration [27].

The success of XEN® hinged on building a multidisciplinary team, developing a patient registry, and integrating high-quality overseas clinical evidence. The multidisciplinary team consisted of RWE research experts, clinical experts, and a professional data collection team [28]. Moreover, the project initiated a patient registry to collect medical information from EMRs, patient-reported outcomes, and follow-ups. The registry also documented the process of data management to ensure data relevance and reliability [28]. Data processing and analyses strictly followed the study protocol, where rigorous domestic RWE was generated. Together with the clinical evidence from overseas registrations, XEN® was approved by the NMPA within 5 months since its application for registration, which was at least seven times faster than the approval process for previous imported innovative medical devices (3 to 5 years) [29].

The approval of XEN® demonstrated preliminarily success of using RWE to support regulatory decision-making in China.

### Uniqueness of Boao Lecheng Pilot Zone

The pilot zone is the only region in China where patients can use innovative medical devices or drugs that have not been approved in China. Patients can benefit from the most state-of-the-art medical treatments without traveling abroad. For examples, in March 2021, the world's first surgery applying Mammotome's dual vacuum-assisted breast biopsy system (the fourth generation of Mammotome) was conducted in the pilot zone; in October 2021, China's first liver cancer clinical treatment operation using yttrium-90 resin microspheres was successfully conducted in Boao Lecheng, meaning Chinese patients with liver cancer can receive the most advanced selective internal radiotherapy domestically [30]. Moreover, there could be a price advantage in the pilot zone, e.g., the Boston Keratoprosthesis implantation costs at least RMB700,000 in the United States, while it only costs RMB100,000 in the pilot zone [30]. The pilot zone has partnered with over 80 medical device companies from 16 countries or regions, introduced 270 innovative medical devices, becoming one of the main channels for imported innovative medical devices to enter China [31].

Moreover, Boao Lecheng is the only “RWD Application Pilot Site” in China, allowing accelerated approvals of international innovative medical devices [31]. Since XEN® was approved in March 2020, eight additional imported innovative medical devices have received registration in China leveraging RWD in the pilot zone [31]. For examples, a laser machine (CATALYS®) for laser cataract surgery was approved in January 2021 [32]; a water vapor therapy (Rezūm™) and its delivery device (Rezum Delivery Device) for benign prostatic hyperplasia were approved in March 2022 [33]. By December 2022, Chinese government has approved RWE research for about 30 medical devices in the pilot zone [31]. The successful experiences in Boao Lecheng could lead RWE research and the use of RWE in regulatory decision-making in China.

### Future of Boao Lecheng Pilot Zone

The current RWE research in “Boao Lecheng Pilot Zone” is still in a preliminary stage. It is used for accelerating the clinical evaluation of medical devices mainly through conducting registries. In the future, the pilot zone aims to build comprehensive databases with data from EMRs, patient-reported outcomes, follow-ups, etc., for specific diseases. The databases could be simultaneously utilized to generate RWE for disease management, medical device evaluation, policy reform, etc. [19].

Moreover, the establishment of “Boao Lecheng Real-World Data Research Center” intents to develop talents and build an outstanding RWE research ecosystem in the pilot zone [28].

The “Hainan Real-World Data Research Platform” will be eventually constructed in two steps: the first step is to develop the “Boao Lecheng Real-World Data Research Platform”; the second step is to establish the “Hainan Real-World Data Research Platform” based on the first step, integrating data sources across the entire Hainan Province, including EMRs, medical claims, registries, etc. Relevant stakeholders could leverage the platform for decision-markings on medical device registration, reimbursement, and health policy, etc. [28].

In conclusion, RWE research in “Boao Lecheng Pilot Zone” is in an initial phase. Stakeholders should institute a more mature system, leading the way on utilizing RWE for market approval of innovative medical devices in China.

## Discussion

This paper outlines the current use of RWE in medical devices in China. Although it is evident that RWD are increasingly involved in the evidence generation of medical devices, we also identified some limitations of its application in China.

The NMPA guideline on the use of RWD for medical device clinical evaluation is still a draft and may be refined in the future. For examples, the NMPA could specify how to conduct real-world studies meeting regulatory standards, as the Food and Drug Administration or the European Medicines Agency [7, 8]; the guideline could also recommend stakeholders to initiate early dialogues with the NMPA, regarding the best approach for regulatory submission utilizing RWE. Medical device stakeholders should continuously monitor the updates of the guideline.

In the past several years, we have observed the integration, standardization, and structurization of data from different information systems within some elite hospitals. However, the disconnection across different hospitals makes the longitudinal follow-up of a patient impossible. The limited data accessibility and data sharing, and the lengthy ethical approval process raise the hurdle of conducting real-world studies in China. Lack of key outcomes for medical devices in EMRs is another challenge to generate RWE for medical devices. To drive the development of RWE research, we need favorable policies and collaboration from relevant stakeholders to encourage data access and sharing, develop systematic technical guidance, and improve data quality.

The pilot zone is pioneering in RWE research in China, which is still in exploratory phase. A small number of patients who need urgent treatments have entered the pilot zone and received care. Normally physicians who can practice in both Boao Lecheng and local hospitals would recommend suitable patients to go. Moreover, companies with medical devices or drugs listed in the pilot zone would advertise the innovative treatments and provide supporting programs, e.g., travel reimbursement. However, limited research has compared patients who entered the pilot zone and those who did not. Patients with severe or rare diseases may be more inclined to try new treatments that can fulfil urgent and unmet medical needs. Patients with moderate-to-high income may be more likely  to travel. Therefore, we need more rigorous research to examine the representativeness and quality of RWE generated in the pilot zone in the future. Overall, the policy environment is evolving towards the use of RWE in China. With the continuing development of the pilot zone, we anticipate observing more accelerated approvals of innovative medical devices and drugs. More patients could receive treatments for urgent and unmet needs.

Another opportunity for the development of RWE research is the large quantity of health-related data benefiting from the large population in China and the high level of digitalization in the healthcare sector. With the application of new technologies (such as cloud computing and machine learning), the healthcare data ecosystem will continue to evolve and improve. Additionally, a massive amount of RWD could be collected from the devices themselves, wearables, and mobile apps.

## Conclusions

Globally, we are witnessing the rising of exciting possibilities for RWE to support clinical evaluation of medical devices. Using RWE to support regulatory decision-making for medical devices is still in an early stage in China, but the guideline issued by the NMPA has encouraged its application. “Boao Lecheng Pilot Zone” provides a unique opportunity for innovative medical devices to gain faster market access to China, leveraging RWD collected there. However, improving data quality is the key success factor, demanding collaborations from government bodies, hospitals, the health care industry, etc. With favorable policy environment and rapid digitalization of every aspect of Chinese’ daily life, RWE will play a more and more important role in improving patients’ health.

## Data Availability

Not applicable.

## Abbreviations

CFDA: China Food and Drug Administration
EMR: Electronic medical record
OPC: Objective performance criteria
NMPA: National Medical Products Administration
RWD: Real-world data
RWE: Real-world evidence
